# Characterization of Full-Length Enterovirus 71 Strains from Severe and Mild Disease Patients in Northeastern China

**DOI:** 10.1371/journal.pone.0032405

**Published:** 2012-03-29

**Authors:** Xiaomei Wang, Chunfeng Zhu, Wanguo Bao, Ke Zhao, Junqi Niu, Xiao-Fang Yu, Wenyan Zhang

**Affiliations:** 1 Institute of Virology and AIDS Research, First Hospital of Jilin University, Changchun, Jilin Province, China; 2 Department of Hepatology, First Hospital of Jilin University, Changchun, Jilin Province, China; 3 Department of Infectious Diseases, First Hospital of Jilin University, Changchun, Jilin Province, China; Faculty of Biochemistry Biophysics and Biotechnology, Jagiellonian University, Poland

## Abstract

Human enterovirus 71 (EV71)-associated hand, foot, and mouth disease (HFMD) has been a leading cause of childhood infection in China since 2008. Epidemic and molecular characteristics of HFMD have been examined in many areas of China, including the central and southern regions. However, clinical and genetic characterization of EV71 in the northeastern region of China is scarce. In this study, a series of analyses were performed on seven full-length EV71 sequences from HFMD patients who had either severe or mild disease. We have determined that these seven circulating EV71 viruses from Changchun, China are actually complex recombinant viruses involving multiple type A human enterovirus (HEV). Classified as EV71 subtype C4 (EV71 C4), these Changchun EV71 viruses contain genetic recombination events between the CA4, CA5, EV71B4 and EV71C1 strains. Most of the structural protein region (P1) of these viruses resembled that of the prototype EV71 C1 strains. The non-structural protein domains (P2 and P3) showed a high degree of similarity with CA4, CA5 and EV71 B4 in different regions. The 5′UTR had unclassified recombination,while partial 3D region of these viruses showed a high degree of similarity to CA16. Phylogenetic analysis of full-length or partial sequences of isolates from severe or mild disease patients in Changchun always formed a single cluster in various phylogenetic analyses of different genomic regions, suggesting that all seven strains originated from one single common ancestor. There was no correlation between viral genomic sequence and virulence. Thus, we found that circulating recombinant forms of EV71 are prevalent among HFMD patients in Northeastern China. The existence of a unique cluster of EV71 related viruses in Northeast China has important implications for vaccine development that would address the increasing prevalence of HFMD.

## Introduction

Hand, foot and mouth disease (HFMD) is a common, mild and self-limiting rash-associated illness in children, with coxsackievirus A16 (CA16) or enterovirus 71 (EV71) as the causative agent [1], [2]. Since first being described in California in 1969 [3], EV71 has been reported to be responsible for many large outbreaks all over the world, including outbreaks that occurred in Malaysia in 1997 [4], Taiwan in 1998 [5], Singapore in 2000 [6], Japan in 1997 and 2000 [2], and Shandong and Fuyang of China in 2007 [7] and 2008 [8], respectively. Generally, the outbreaks were associated with severe neurologic disease, such as acute flaccid paralysis, pulmonary edema, myocarditis, and fatal encephalitis.

EV71 is a member of the human enterovirus group A (HEV-A) and contains a positive, single-stranded RNA genome of approximately 7500 bases and a single open reading frame followed by a poly A tract [9], [10]. The viral genome contains 5′- and 3′-untranslated regions (UTRs) that are essential for viral expression and replication. The genome encodes for a single, large polyprotein that is composed of four capsid proteins, VP1 to VP4, and seven nonstructural proteins, 2A, 2B, 2C, 3A, 3B, 3C, and 3D [9]. The EV71 viruses were classified into three independent lineages, A, B, and C, based on the structural VP1 gene; each group has at least 15% divergence from the others [11]. Group A consists of one member, the prototype BrCr strain. The B group, which has been predominant in Malaysia and Singapore, was separated into subgroups B1 to B5. The C group, which has been predominant in east Asia, contained subgroups C1 to C5 [12]. EV71 has a high mutation rate due to low-fidelity replication and frequent recombination [13].

Since the 1980s, large and small EV71 epidemics caused by distinct genotypes have occurred in Asian countries and in regions sharing trade with China [13]. EV71 epidemics have been reported in Jilin Province since 2006, and the incidence of HFMD has increased annually. Here, we report the clinical and molecular characteristics of EV71-infected patients from Changchun, China in 2010. The complete genomes of seven EV71 strains from mild and severe patients were sequenced and analyzed along with summarized clinical information. The results of this study demonstrated that a combination of intratypic and intertypic recombination involving multiple HEV-A strains occurred within all seven Changchun EV71 sequences. This suggests that recombination may be one of the potential reasons for persistent infection and emerging outbreaks in China. Moreover, all seven Changchun strains always formed a single cluster with a high bootstrap value in various phylogenetic analyses of different genomic regions, suggesting that all seven strains originated from one single common ancestor, regardless of whether they occurred in fatal or non-fatal cases; thus, we could not see any correlation between genome and virulence.

## Results

### The clinical characteristics of seven patients

Seven throat swabs were chosen from 84 patients who were identified as EV71-positive by a diagnostic kit (DAAN Gene Co., Ltd. of Sun Yat-Sen University). These seven patients from whom the EV71 was identified were young children (median age of 23 months; ranged from 7 months to 5 years): three were males, and four were females. All clinical information was obtained from the medical records of the First Hospital of Jilin University and is summarized in Table 1. All patients presented with HFMD, manifested by fever, oral ulcers and vesicular on the hand, and in some cases, buttocks and knees. Four cases, including those associated with mild central nervous system (CNS) symptoms such as vomiting, myoclonic jerk, and irritability, were cured after an average of 6 days of hospitalization. One case (Changchun063) was complicated by irregular respiration, tachypnea (50 times/min) and encephalitis, which resulted in placing the patient on a ventilator; the patient subsequently recovered after 8 days of treatment. Two cases (Changchun014 and103) were complicated by tachypnea, tachycardia, hypotension, pulmonary edema, severe oliguria and encephalitis; these complications proved fatal in the patients after 2 days of hospitalization.

**Table 1:** Clinical manifestations of 7 enterovirus 71 infected patients.

No. of Sample	Changchun011	Changchun014	Changchun063	Changchun072	Changchun077	Changchun103	Changchun128
Sex	Male	Female	Male	Male	Female	Female	Female
Age	5Y	1Y	7M	3Y	2Y	11M	9M
Clinical manifestation	Fever	Fever	Fever	Fever	Fever	Fever	Fever
	Skin vesicular	Tachycardia	Skin vesicular	Skin vesicular	Skin vesicular	Skin vesicular	Skin vesicular
	Oral ulcer	Hypotension	Oral ulcer	Oral ulcer	Oral ulcer	Oral ulcer	Oral ulcer
		Lethargy	Irregular respiration	Vomiting	Irritability	Myoclonic jerk	
		Oliguria	Tachypnea	Myoclonic jerk		Lethargy	
				Irritability		Hypotension	
						Tachypnea	
						Tachycardia	
Complication	No	Encephalitis	Encephalitis	No	No	Encephalitis	No
		Heart failure					
		Pulmonary edema					
Diagnosis	HFMD	HFMD	HFMD	HFMD	HFMD	HFMD	HFMD
Days of fever	2	2	4	3	3	3	2
Days of hospitalization	6	2	8	5	8	2	4
Underlying disease	No	No	No	No	No	No	No
Outcome	Recovery	Death	Recovery	Recovery	Recovery	Death	Recovery

Y = Year, M = Month.

**Figure 1:**
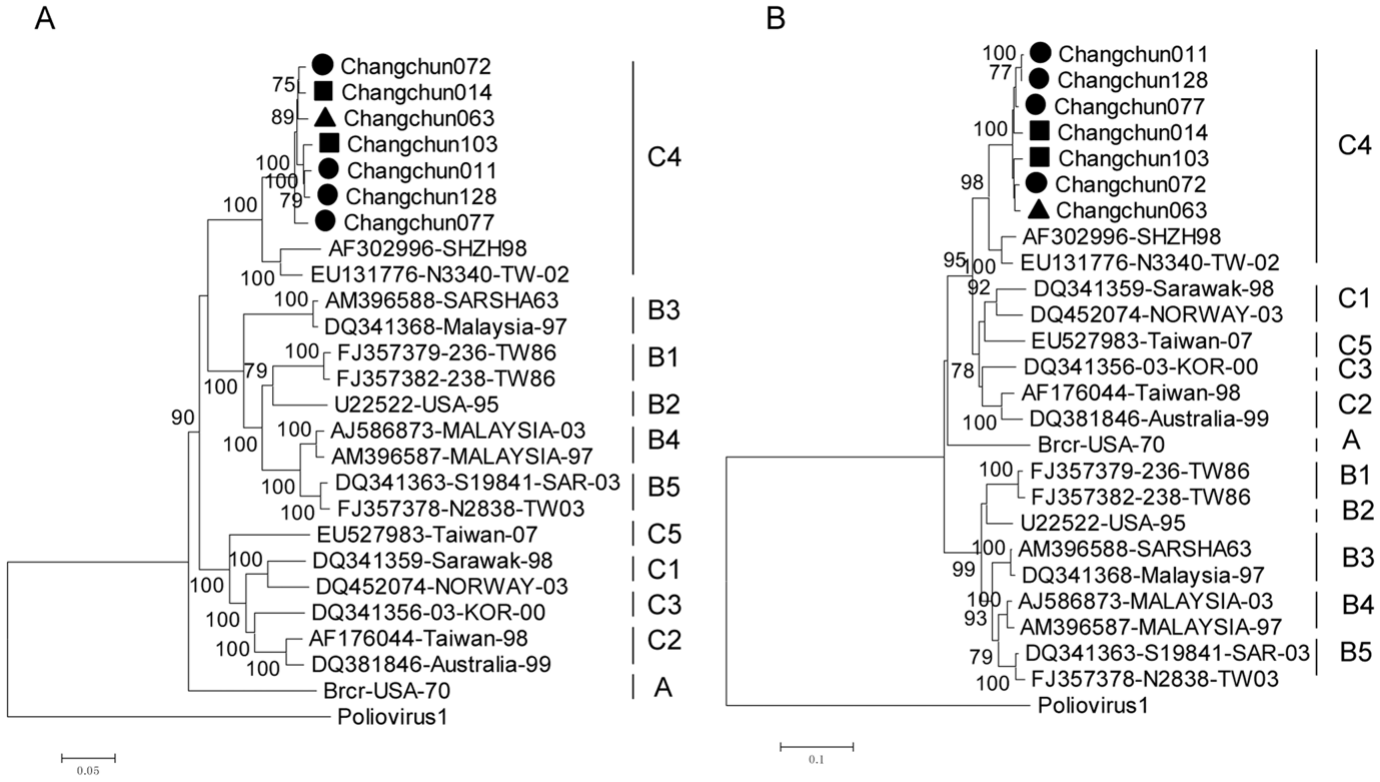
Phylogenetic analysis of the complete genome and VP1 protein-coding region of the Changchun strains. Phylogenetic trees were generated by the neighbor-joining method with 1000 bootstraps for 7 representative Changchun strains and other EV71 strains of known subgenotypes. The poliovirus 1 strain was used as the outlier. The ▪ icon indicates the fatal cases; ▴ indicates the severe cases; • indicates the mild cases. A: Phylogenetic tree based on the whole genome sequences. B: Phylogenetic tree based on the VP1 region (891 bp).

Using the poliovirus 1 sequence as an outlier, a phylogenetic analysis of the complete genome sequences for the HEV-A strains with these seven samples revealed that the seven Changchun viruses were clustered into the EV71 C4 subtype; this subtype was more closely related to subtype B but not to subtype C (Fig. 1A), although the bootstrap value was 60, lower than 70, which could not be considered significant. The phylogenetic tree based on the VP1 gene sequence showed that the seven Changchun strains were clustered into the subtype C4 lineage; which closely related to subtype C in this region (Fig. 1B). The incongruent phylogenetic relationships observed between the complete genome and VP1 sequences suggest that possible recombination events had occurred in the seven EV71 and SHZH98 strains.

### Seven EV71 isolated from HFMD patients in Changchun are circulating, recombinant strains

**Figure 2:**
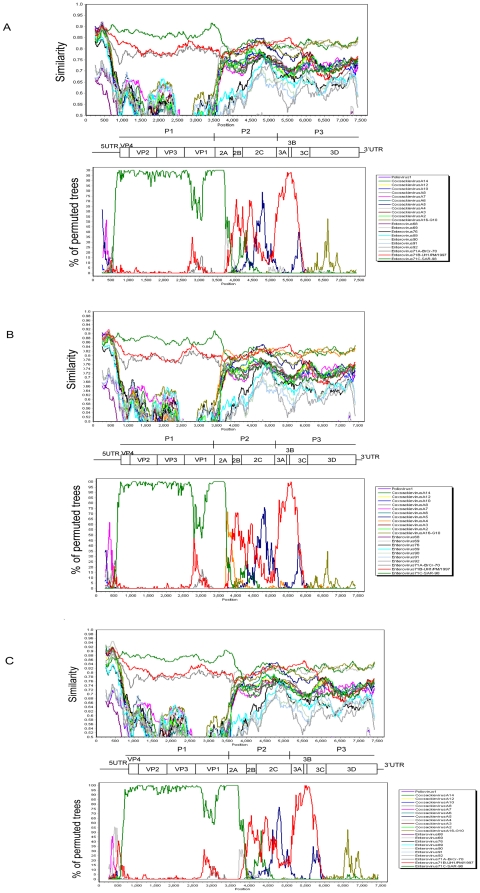
Identification of recombination in the Changchun strains. A: Similarity plot and bootscan analysis for Changchun011. B: Similarity plot and bootscan analysis for Changchun077. C: Similarity and bootscan analysis for Changchun103 (others not shown). A window size of 500 nucleotides in increments of 20 nucleotides at a time was used. Positions containing gaps were not excluded from the comparison.

To further characterize the genomic features of the seven strains isolated from Changchun in 2010, each Changchun representative strain of EV71 was analyzed by similarity plot and bootscan analysis against other enteroviruses from HEV-A as reference sequences. Strains BrCr, AM396587-UH1PM1997 and DQ341359-SAR-98 were selected to represent EV71 type A, B and C, respectively, while poliovirus 1 was used as an outlier. EV68 was also included as another outlier to enhance the results. As a result, all Changchun EV71 genomes displayed intertypic and intratypic recombination involving multiple type A HEV (See Fig. 2, showing the Changchun011 strain from a mild case without CNS symptom, the Changchun077 strain from the CNS symptoms case and the Changchun103 strain from a fatal case). The recombination patterns of the other four strains are similar to those of Changchun011 (Fig. 2A) and 103 (Fig. 2C). Only Changchun077 had a specific recombinant involving CA4 among P2 region. In the similarity plot analysis, the sequences of the seven EV71 strains showed high similarity (77%–100%) to the EV71 genotype C strain (DQ341359-SAR-98) based on the P1 region. Low similarity (71% and 86%) to the EV71 genotype B and CA5 strains was alternatively noted in the Changchun EV71 strains among the P2 and partial P3 regions. Before position 500 and after position 5860, locations that correspond to the 5′UTR and 3D regions, respectively, the seven EV71 strains showed no significant similarity to any reference sequence. In the bootscan analysis, the results again indicated that P1 regions of all seven Changchun sequences were originated from EV71 type C. In the following analysis of the P2 and partial P3 regions, however, high bootstrap values showed that the seven EV71 strains contained alternative regions that were closely related to the EV71 genotype B and CA5 strains. For the 3D region, bootscan analysis did not support any clustering of the seven EV71 strains. These findings indicated that intertype and intratype recombination events occurred in the P2 and P3 regions.

### Further analysis of the 5′UTR and 3D regions

**Figure 3:**
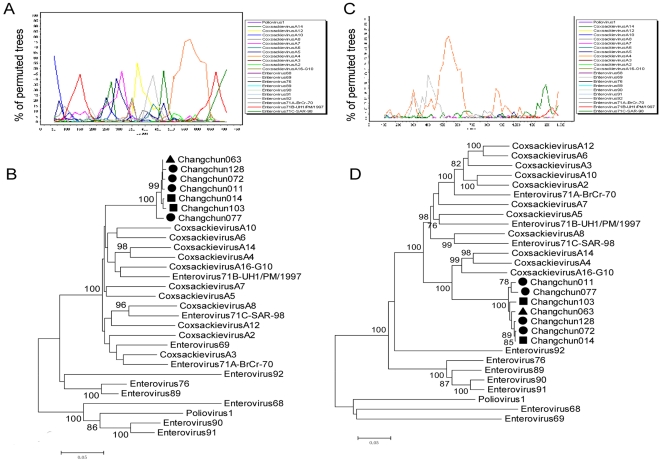
Bootscan and phylogenetic analyses of partial genomes from the Changchun strains. A: Identification of the recombinant sequences in the 5′-UTR region of the Changchun011 genome. The window size of 100 nucleotides slides in increments of 20 nucleotides at a time. B: The neighbor-joining tree was established from alignments of the entire 5′-UTR region for the seven Changchun strains. C: Bootscan analysis of a 3D region in the Changchun011 genome. The window size included 200 nucleotides slides in increments of 20 nucleotides at a time. D: The neighbor-joining tree was established from sequence alignments of the entire 3D region for the seven Changchun strains. The ▪ icon indicates the fatal cases; ▴ indicates the severe cases; • indicates the mild cases.

The results described previously showed that no obvious parental sequence was found for 5′UTR and 3D regions of the seven Changchun EV71 strains, therefore, we carefully examined the 5′UTR and 3D regions by bootscanning and phylogenetic tree analyses. A more detailed bootscanning of the 5′UTR of the seven strains with a smaller window of 100 bp was performed, and this analysis showed a low bootstrap with any reference sequence (Fig. 3A). Phylogenetic analysis of the 744-bp fragment of the 5′UTR region is consistent with the bootscanning results, showing that the sequence did not form a cluster with any specific virus (Fig. 3B). A more detailed bootscanning of the 3D region within the seven strains using a smaller window of 200 bp was performed; it showed a dominant CA16 sequence in the middle (Fig. 3C). Phylogenetic analysis of the 1386-bp fragment of the 3D region from the seven EV71 strains is consistent with the bootscanning results, showing a single cluster that was closely related to CA16 (Fig. 3D).

### Further analysis of the P1, P2, 2A and 3C regions

**Figure 4:**
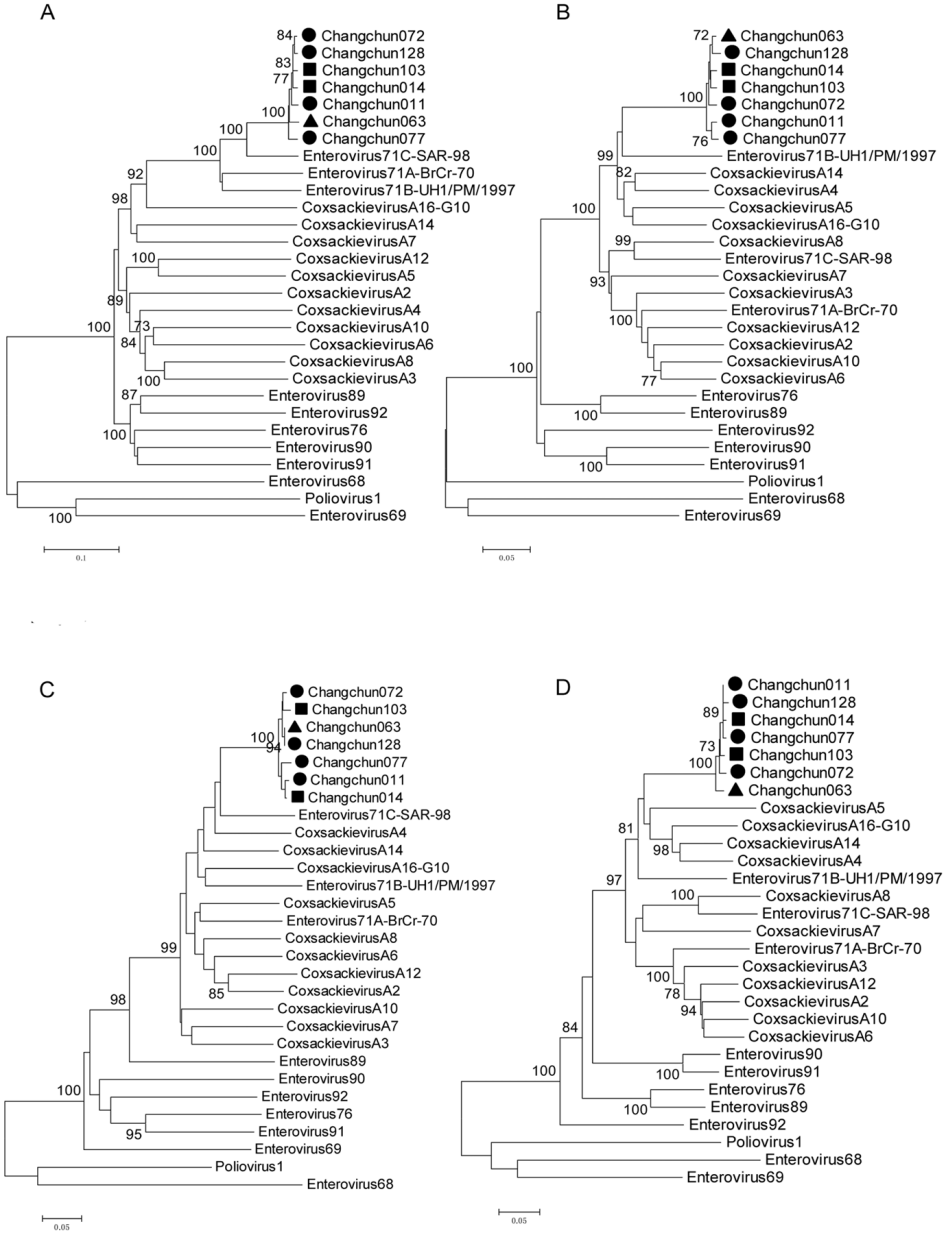
Phylogenetic analysis for partial genomes from the Changchun strains. A: Based on the entire P1 gene region of the Changchun strains, B: Based on the P2 region of the Changchun strains, C: Based on the 2A region of the Changchun strains, D: Based on the 3C region of the Changchun strains The ▪ icon indicates the fatal cases; ▴ indicates the severe cases; • indicates the mild cases.

We further analyzed the phylogenetic trees of P1 (VP4-VP1), P2 (2A–2C) and other regions of the seven Changchun strains. Phylogenetic analysis showed that the seven Changchun strains were clustered with the EV71 genotype C strains for P1 region (Fig. 4A), but with EV71 genotype B strains for P2 region (Fig. 4B). The 2A region of the Changchun strains was related to the sequence of the EV71 genotype C strains (Fig. 4C). The 2C and 3A regions of the strains were closely related to CA5 and EV71B respectively (data not shown), although the bootstrap value was very low. Moreover, the 3C region of the Changchun strains was related to the sequence of CA5 (Fig. 4D). These results further confirmed that the Changchun strains are complex recombinants involving multiple HEV-A strains.

### Phylogenetic analysis of the seven Changchun strains with other strains in China

**Figure 5:**
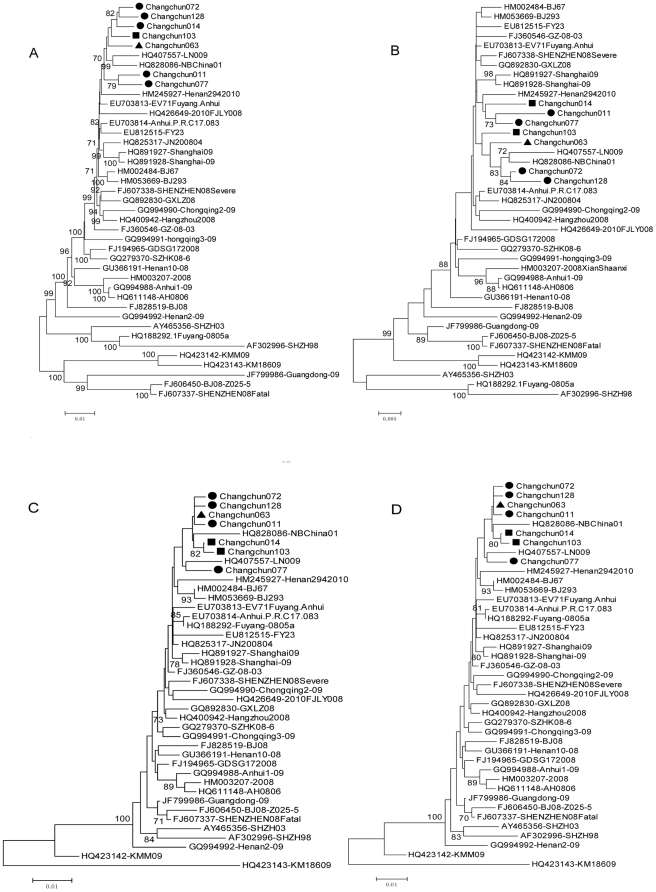
Phylogenetic analysis of seven Changchun strains and GenBank database sequences of the EV71 strains from other provinces in China. A: Based on the complete genome sequence, B: Based on the VP1 region sequence, C: Based on the 5′-UTR region sequence, D: Based on the P2 region sequence,.. The ▪ icon indicates the fatal cases; ▴ indicates the severe cases; • indicates the mild cases.

To assess and compare the molecular characteristics of the seven Changchun isolates with the EV71 strains from the other provinces in mainland China, phylogenetic trees were constructed using the complete genome as well as partial regions (Fig. 5). The outlier group was not used here because all of the China strains used here were clustered as subtype C4. GenBank database sequences of 34 epidemic strains from Shenzhen, Henan, Fuyang and other regions of China were included for comparison. When comparing the complete genome (Fig. 5A), structural protein VP1 genes (Fig. 5B), 5′-UTR (Fig. 5C) nonstructural protein P2 genes (Fig. 5D) and region phylogenetic trees, the likelihood of Changchun strains clustering with the LN009 (Liaoning), NBChina01 (Ningbo) and Henan2 strains revealed the possible evolution of Changchun strains from these three reference strains. However, according to the phylogenetic analysis, strains from both severe and mild HFMD patients kept mixing together, thus it was hard to determine the relationship between viral sequence and virulence. Interestingly, the 3C regions of the majority of EV71 strains formed a large cluster (data not shown). We inferred that, as a protease, 3C protein has to precisely recognize cutting sites within the polyprotein; thus, 3C has to be conservative to maintain the conformation.

## Discussion

Molecular characterizations of circulating strains in central and southern China have been well studied [8], [14], [15]. However, little has been reported on the genetic characteristics of EV71 strains circulating in the northeastern region of China. In this study, we examined for the first time seven EV71 strains isolated from severe- or mild-diseased patients in 2010 in Changchun by phylogenetic tree, bootscan and similarity plot analyses. C4 was previously identified as the most prominent EV71 subgenotype circulating in China [15]. Phylogenetic analyses on the complete genome and VP1 sequences showed that all Changchun strains belong to the C4 subgenotype (Fig. 1). This result suggests that Changchun EV71 did not evolve independently. Complete genome sequences were closely related to EV71 subtype B, while VP1 sequences were closely related to EV71 subtype C. The incongruent phylogenetic relationships suggest that the recombination event has occurred.

Many reports have described recombination in enteroviruses as a general phenomenon between different types of enteroviruses of the same species [8], [16], [17], [18], [19] Further analyses of the full-length genomic sequences of the seven isolates by similarity plot and bootscan analysis showed that genetic recombination events existed between CA4, CA5, CA16, EV71B and EV71C strains (Fig. 2). The 5′UTR region of the seven Changchun strains were unclassified due to a low sequence homology to any reference strain. The 3D region of the seven strains had the highest similarity to CA16 (Fig. 3). Most of the structural protein (P1) region resembled that of the prototype EV71 type C. The non-structural protein domains (P2 and P3) showed a high degree of similarity with CA4, CA5 and EV71 type B in different regions. Recombination appeared to be frequent in the P2 and P3 regions but rare in the P1 region, probably because of the structural constraint that exists to properly assemble the viral capsid. The non-structural regions, P2 and P3, are likely the hot spots for recombination in enteroviruses. Our present findings are consistent with a previous study demonstrating, through genomic analysis, that several recombination breakpoints were located within the P2 and P3 regions of enteroviruses of the same species, while no breakpoints were found within the P1 regions. However, the seven Changchun strains consistently clustered together in all of the analyzed regions, suggesting all these EV71 sequences had a single common ancestor, and kept circulating and evolving within Jilin Province.

Moreover, genetic recombination of these seven Changchun strains was similar to the SHENZHEN-98 strain first classified as subtype C4 in mainland China by bootscan analysis (data not shown). Genetic recombination could result in the emergence of viruses with altered pathogenic potential, which could cause a serious public health threat for children because inventing a vaccine for a rapidly mutating virus is very difficult. There is an urgent need to establish an effective HEV71 surveillance system to tackle the increasing threat that these viruses pose in China.

Phylogenetic analysis on full-length or partial sequences from isolates obtained from severe or mild disease patients in Changchun did not reveal any clustering with EV71 viruses circulating in China (Fig. 5). Li et *al* [20] described several sites important for virulence that are not shown in these seven Changchun strains from fatal and non-fatal cases. Additionally, there was no relationship between the genome sequences from severe or mild patients and fatality. Further studies will be required to identify viral determinants of severe HFMD in Northeast China.

## Materials and Methods

### Ethics Statement

This study has obtained ethics approval from the ethics committee at the First Hospital of Jilin University. Written informed consent was obtained from the parents of all the children involved in our study.

### Information of seven EV71 patients

Seven EV71 strains were chosen from 84 throat swab samples obtained from HFMD patients in 2010; the EV71 strains were identified with real-time PCR. Of the seven EV71 strains, the Changchun011 and Changchun128 strains were from patients with mild symptoms, such as slight fever, oral ulcers and skin rashes on their palms and soles; Changchun014 and Changchun103 strains were from patients who died; Changchun063 strains were from patients with severe complications that survived; Changchun072 and Changchun077 strains were from patients who exhibited typical clinical symptoms of CNS involvement, such as fever, vomiting and myoclonic jerking. All the patients were hospitalized at the First Hospital of Jilin University.

### Reverse transcription PCR

Viral RNA was extracted from 200 µl of throat swabs using TRIzol (Invitrogen). The cDNA was generated using the High-Capacity cDNA Reverse Transcription Kit (Applied Biosystems) and oligo-dT primers according to the supplier's instructions. Nine pairs of overlapping primers were designed according to the conserved regions of the Anhui fuyang, Chongqing1, Henan1, Shenzhen98 strains. The PCR parameters for all the primer pairs were as follows: cDNA were denatured at 94°C for 4 min. The amplification was performed in 35 cycles consisting of a denaturing step for 30 s at 94°C, a primer annealing step for 30 s at 50°C to 56°C, and a two-part elongation step for 1 to 2 min at 72°C, then extended at 72°C for 5 to 8 min. The reactions were analyzed by electrophoresis on 1.0% agarose gels.

### Nucleotide sequencing

Amplifications were either sequenced directly or purified with an E.Z.N.A.Gel Extraction Kit (OMEGA), cloned into the pGEM-T Easy vector (Promega, USA) and sequenced with T7 and SP6 primers. All sequencing was performed by Sangon Biotech (Shanghai Co., Ltd.) using the BigDyeterminatorv3.1 kit and ABI-PRISM3730XL DNA sequencer (Applied Biosystems, USA).

The EV71 full-length genomes were acquired by assembling all of the fragments using the DNAMAN5.2.2 software.

### Phylogenetic analysis

The alignments of the seven Changchun EV71 strains and reference sequences were achieved with the MEGA4 program and Clustal W software. Phylogenetic and molecular evolutionary analyses were conducted using the neighbor-joining method and Kimura 2-parameter model with 1000 bootstraps pseudoreplicated with the MEGA5 program [15], [21]. Bootstrap values lower than 70% were hidden. The length of nucleotides used for the analysis varied, depending on the purpose of the particular analysis, which is clearly indicated in the Results section.

### Recombination analyses

To analyze bootscanning and nucleotide similarity between Changchun EV71 and other HEV genomes, the sequence alignments were first completed with MEGA4.1. The results were then analyzed using bootscan analysis in SimPlot, version 3.5.1. The neighbor-joining method and Kimura 2-parameter model were selected for all bootscanning [22]. The window and step sizes were determined based on the intent of the analysis and the length of the sequences. The reference sequences were CA2, 3, 4, 5, 6, 7, 8, 10, 12, 14, and 16 prototype strains, the poliovirus1 strain, human enterovirus 68, 69, 76, 89, 90, 91, and 92 prototype strains, the EV71 A prototype strain, BrCr, the B4 genotype representative strain, AM396587-UH1PM1997 and C1 genotype representative strain DQ341359 -SAR-98.

## References

[1] Chen K-T, Chang H-L, Wang S-T, Cheng Y-T, Yang J-Y (2007). Epidemiologic Features of Hand-Foot-Mouth Disease and Herpangina Caused by Enterovirus 71 in Taiwan, 1998 2005.. Pediatrics.

[2] Shimizu H, Utama A, Onnimala N, Li C, Li-Bi Z (2004). Molecular epidemiology of enterovirus 71 infection in the Western Pacific Region.. Pediatr Int.

[3] Schmidt NJ, Lennette EH, Ho HH (1974). An apparently new enterovirus isolated from patients with disease of the central nervous system.. J Infect Dis.

[4] Chumakov M, Voroshilova M, Shindarov L, Lavrova I, Gracheva L (1979). Enterovirus 71 isolated from cases of epidemic poliomyelitis-like disease in Bulgaria.. Arch Virol.

[5] Ho M, Chen ER, Hsu KH, Twu SJ, Chen KT (1999). An epidemic of enterovirus 71 infection in Taiwan. Taiwan Enterovirus Epidemic Working Group.. N Engl J Med.

[6] Chan KP, Goh KT, Chong CY, Teo ES, Lau G (2003). Epidemic hand, foot and mouth disease caused by human enterovirus 71, Singapore.. Emerg Infect Dis.

[7] Zhang Y, Tan XJ, Wang HY, Yan DM, Zhu SL (2009). An outbreak of hand, foot, and mouth disease associated with subgenotype C4 of human enterovirus 71 in Shandong, China.. J Clin Virol.

[8] Zhang Y, Zhu Z, Yang W, Ren J, Tan X (2010). An emerging recombinant human enterovirus 71 responsible for the 2008 outbreak of Hand Foot and Mouth Disease in Fuyang city of China.. Virology Journal.

[9] Chan Y-F, Sam IC, AbuBakar S (2010). Phylogenetic designation of enterovirus 71 genotypes and subgenotypes using complete genome sequences.. Infection, Genetics and Evolution.

[10] Chang G-h, Lin L, Luo Y-j, Cai L-j, Wu X-y (2010). Sequence analysis of six enterovirus 71 strains with different virulences in humans.. Virus Research.

[11] Brown BA, Oberste MS, Alexander JP, Kennett ML, Pallansch MA (1999). Molecular epidemiology and evolution of enterovirus 71 strains isolated from 1970 to 1998.. J Virol.

[12] Solomon T, Lewthwaite P, Perera D, Cardosa MJ, McMinn P (2010). Virology, epidemiology, pathogenesis, and control of enterovirus 71.. Lancet Infect Dis.

[13] Li L, He Y, Yang H, Zhu J, Xu X (2005). Genetic characteristics of human enterovirus 71 and coxsackievirus A16 circulating from 1999 to 2004 in Shenzhen, People's Republic of China.. J Clin Microbiol.

[14] Zhu Z, Xu WB, Xu AQ, Wang HY, Zhang Y (2007). Molecular epidemiological analysis of echovirus 19 isolated from an outbreak associated with hand, foot, and mouth disease (HFMD) in Shandong Province of China.. Biomed Environ Sci.

[15] Mao L-X, Wu B, Bao W-X, Han F-a, Xu L (2010). Epidemiology of hand, foot, and mouth disease and genotype characterization of Enterovirus 71 in Jiangsu, China.. Journal of Clinical Virology.

[16] Yoke-Fun C, AbuBakar S (2006). Phylogenetic evidence for inter-typic recombination in the emergence of human enterovirus 71 subgenotypes.. BMC Microbiol.

[17] Shi X, Jin Q, Hu Y, Chi X, Gao Y (2011). Dyslipidemia in northeastern China.. Central European Journal of Medicine.

[18] Huang SC, Hsu YW, Wang HC, Huang SW, Kiang D (2008). Appearance of intratypic recombination of enterovirus 71 in Taiwan from 2002 to 2005.. Virus Res.

[19] Chan YF, AbuBaker S (2004). Recombinant human enterovirus 71 in hand, foot and mouth disease patients.. Emerg Infect Dis.

[20] Li R, Zou Q, Chen L, Zhang H, Wang Y (2011). Molecular Analysis of Virulent Determinants of Enterovirus 71.. PLoS One.

[21] Tan X, Huang X, Zhu S, Chen H, Yu Q (2011). The Persistent Circulation of Enterovirus 71 in People's Republic of China: Causing Emerging Nationwide Epidemics Since 2008.. PLoS One.

[22] Huang SW, Hsu YW, Smith DJ, Kiang D, Tsai HP (2009). Reemergence of enterovirus 71 in 2008 in taiwan: dynamics of genetic and antigenic evolution from 1998 to 2008.. J Clin Microbiol.

